# Prehabilitation: preoperative rehabilitation interventions for lung cancer – a scoping review

**DOI:** 10.3389/fragi.2025.1665955

**Published:** 2025-10-15

**Authors:** Ana Jesus Colaço, Cidália Castro, Steven Hall, Júlio Belo Fernandes

**Affiliations:** ^1^ Lung and Neuro-Oncology Unit, Champalimaud Foundation, Lisbon, Portugal; ^2^ Egas Moniz Center for Interdisciplinary Research (CiiEM), Egas Moniz School of Health & Science, Almada, Portugal; ^3^ Nurs* Lab, Almada, Portugal; ^4^ Faculty of Nursing, University of Alberta, Edmonton, AB, Canada

**Keywords:** lung cancer, prehabilitation, preoperative period, rehabilitation nursing, Physioterapy

## Abstract

**Background:**

Individuals undergoing lung cancer surgery often face significant postoperative challenges, underscoring the importance of identifying effective preoperative rehabilitation strategies to support recovery.

**Aim:**

To identify rehabilitation interventions that can be implemented during the preoperative period for individuals with lung cancer undergoing thoracic surgery.

**Design:**

Scoping review guided by the Arksey and O'Malley methodological framework.

**Methods:**

The research question guiding this review was “What rehabilitation interventions should be implemented in the preoperative period for individuals with lung cancer undergoing surgery?” A comprehensive search was performed across five databases: MEDLINE, Cochrane Central, CINAHL, ScienceDirect, and PubMed. The review included studies that addressed rehabilitation interventions before thoracic surgery for individuals with lung cancer.

**Results:**

A total of 19 articles met the inclusion criteria. The findings indicate that combining aerobic endurance, resistance, and respiratory training with preoperative education improves outcomes. In addition, nutritional counseling and brief relaxation/emotion-regulation strategies appear to be valuable components of multimodal prehabilitation programs, though evidence is limited.

**Conclusion:**

Preoperative rehabilitation interventions have the potential to enhance functional reserve, reduce postoperative complications, and accelerate recovery in individuals undergoing lung resection for lung cancer.

## 1 Introduction

Lung cancer was the second most prevalent cancer worldwide in 2020, as well as the leading cause of cancer-related death (Sung et al., 2021). Several treatment options are available for this disease; however, surgical resection is an intervention with a favorable prognosis when patients are eligible (Goldsmith et al., 2021; Machado et al., 2023). The indication for surgery in lung cancer cases is expected to increase by approximately 60% by 2040 (Perera et al., 2021). The goal of the surgical procedure is to achieve adequate tumor resection while preserving viable lung tissue (Rotman et al., 2015; Tegegne et al., 2021). Although pulmonary resection improves survival rates, it is associated with significant postoperative complications that affect the quality of life, including physical limitations such as pain, fatigue, and dyspnea (Machado et al., 2023; Motono et al., 2021; Pu et al., 2021; Van et al., 2018). These complications are the primary causes of morbidity and mortality, leading to prolonged hospitalization and increased healthcare costs (Tegegne et al., 2021; Fernandes et al., 2019).

International Enhanced Recovery After Surgery (ERAS) guidelines were established to minimize complications associated with surgical procedures (Batchelor et al., 2019). These guidelines incorporate multimodal, evidence-based interventions during the preoperative, intraoperative, and postoperative periods to shorten hospital stays, reduce postoperative complications, and lower associated healthcare costs (Machado et al., 2023; Van et al., 2018; Batchelor et al., 2019; Mendes et al., 2018). According to these recommendations, in the preoperative period, which is the focus of this review, the ERAS program emphasizes patient education, stress reduction, pain management optimization, physical exercise, nutrition, and improving functional status (Batchelor et al., 2019; Mendes et al., 2018; Haywood et al., 2020; Montagne et al., 2021). This approach is referred to as *prehabilitation*.

Prehabilitation, an emerging concept, can involve either unimodal or multimodal approaches tailored to each person’s individual needs (Silver, 2015; Silver and Baima, 2013; Carli et al., 2017). It is a process within the continuum of care between the moment of diagnosis and the initiation of treatment. This process includes physical and psychological assessments to establish baseline functional levels, identify needs, and provide specific interventions to improve the person’s health. The goal is to reduce the incidence and severity of current and future health issues (Silver, 2015; Silver and Baima, 2013; Carli et al., 2017). In the surgical context, prehabilitation aims to enhance preoperative functional reserve, leading to improved and faster postoperative recovery and a reduction in complications (Pu et al., 2021; Carli et al., 2017).

By enhancing individuals' functional capacity and encouraging active involvement in their own care, prehabilitation has demonstrated positive outcomes for both patients and healthcare systems. This proactive approach not only prepares people physically and psychologically for surgery but also contributes to more efficient hospital management—facilitating smoother patient flow, reducing the need for critical care beds, and shortening hospital stays.

Recent studies have reported improved postoperative outcomes, including in individuals undergoing cancer surgery, highlighting benefits at multiple levels, such as reduced complication rates, quicker recoveries, more efficient care transitions, and a significant reduction in hospital length of stay (Daniels et al., 2020; Gillis et al., 2018; Hughes et al., 2019; Moran et al., 2016).

Two recent syntheses have consolidated the evidence base: a review of exercise-based prehabilitation in people with non-small cell lung cancer (Granger and Cavalheri, 2022) and an overview of reviews focusing on exercise across the lung cancer care continuum (Edbrooke et al., 2023). Building on these contributions, this scoping review adopts a complementary, practice-oriented lens to map preoperative prehabilitation components in adults with lung cancer scheduled for thoracic surgery, detailing what is delivered and how it is delivered, so that readers can clearly understand the interventions in practice. Accordingly, this review aims to identify preoperative rehabilitation interventions for individuals with lung cancer undergoing thoracic surgery and to describe their content and delivery to inform clinical implementation.

## 2 Methods

Scoping reviews are most appropriate when the aim is to identify and map characteristics or concepts across studies and to report and discuss these features, rather than to answer a single, narrowly framed question (Munn et al., 2018). In line with this, we selected a scoping review to map what is delivered and how it is delivered in preoperative prehabilitation for lung cancer surgery across different studies and reporting formats, enabling comprehensive charting of the literature to inform implementation.

This scoping review was conducted using the methodology of Arksey and O’Malle (2007), which comprises five stages. This approach was chosen for its flexibility and adaptability, allowing tailoring to the review’s specific aims (Arksey and O’Malley, 2007). Methods are reported in accordance with the PRISMA-ScR statement (Haddaway et al., 2022). No protocol for this scoping review was publicly registered.

### 2.1 Stage 1: identifying the research question

The research question was formulated using the PCC mnemonic (Population, Concept, Context) to align with the study objectives and inclusion/exclusion criteria (Arksey and O’Malley, 2007). The formulated question guiding this scoping review was: “What rehabilitation interventions (Concept) should be implemented in the preoperative period (Context) for individuals with lung cancer undergoing surgery (Population)?”

### 2.2 Stage 2: identifying relevant studies

The search was conducted between March 20 and 22, 2024, and updated on 8 de September 2025, using the EBSCOhost interface across the following databases: MEDLINE Complete, Cochrane Central Register of Controlled Trials, CINAHL Complete, and Nursing & Allied Health Collection: Comprehensive. Additionally, ScienceDirect and PubMed were also searched. These databases were chosen due to their high indexing of literature related to cancer, rehabilitation medicine, and medical interventions.

Medical Subject Headings (MeSH) terms were used to develop the following search string: ((Lung cancer OR Lung neoplasms OR Lung tumor) AND (Prehabilitation OR preoperative exercise OR rehabilitation OR exercise) AND (Perioperative period OR preoperative care OR preoperative period)). The inclusion and exclusion criteria are summarized in Table 1.

**TABLE 1:** Inclusion and exclusion criteria.

Criteria	Inclusion criteria	Exclusion criteria
Population	People with lung cancer Adult (≥18 years)	Non-oncological pulmonary pathology Age ≤18 years
Concept	Rehabilitation interventions	Studies that do not include rehabilitation interventions
Context	Preoperative period	Interventions delivered outside the preoperative period
Type of studies	Research studies - randomized and quasi-experimental studies	Other types of studies

The criteria also restricted inclusion to full-text articles published between 2014 and 2025 in English or Portuguese. The date limiter was applied to ensure the inclusion of studies reflecting current surgical advancements and perioperative care strategies. Given the continuous evolution of lung resection techniques and the growing emphasis on prehabilitation in surgical recovery, we aimed to capture the most relevant and up-to-date evidence aligning with contemporary clinical practice.

### 2.3 Stage 3: study selection

**FIGURE 1:**
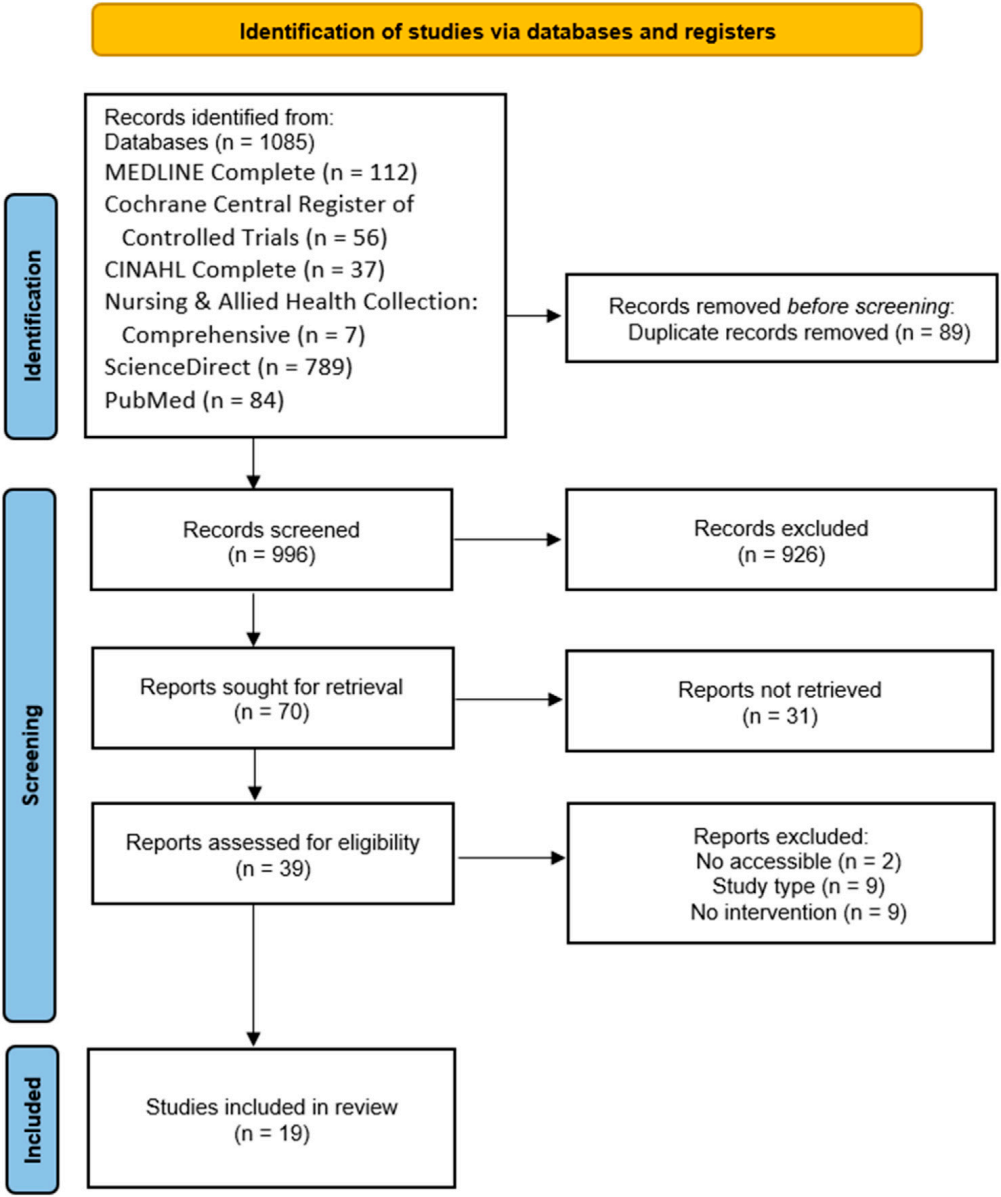
PRISMA flowchart.

The PRISMA flowchart (Haddaway et al., 2022) in Figure 1 demonstrates the identification, screening, and selection process. A total of 1085 articles were identified across the different databases. 89 duplicates were removed, and 926 titles and abstracts were screened. This was followed by an assessment of 39 full texts, finally resulting in 19 included records.

At this stage, Mendeley was used for reference management, while a Microsoft Excel spreadsheet was created to input the references of the retrieved articles. This allowed for clear visualization, systematic screening, and selection according to the predefined eligibility criteria. XXX and XXX completed dual screening of all records.

### 2.4 Stage 4: charting the data

Data extraction synthesizes the information obtained within the scope of the research objective, making it easier to identify essential components. Authors XXX and XXX performed data extraction following the guidelines of Arksey and O’Malley (2007) using a Microsoft Excel table to organize key data items. Extracted items included author(s), year of publication, study location, objectives, study design, and intervention details (activities, characteristics, and descriptions).

### 2.5 Stage 5: collection, summarizing and reporting the results

A thematic analysis of the included articles was conducted, following the six-phase approach of Braun and Clarke (2006), to synthesize the intervention content reported across studies. (1) Familiarization: two reviewers read full texts and extracted verbatim descriptions of interventions. (2) Generating initial codes: working primarily at a semantic level, and where appropriate at a latent level, reviewers independently coded extracts into an evolving codebook. (3) Searching for themes: codes were iteratively clustered into higher-order categories representing meaningful components of prehabilitation. (4) Reviewing themes: candidate themes were checked against coded extracts and the full dataset; boundaries were refined to avoid overlap and to ensure internal coherence. (5) Defining and naming themes: we produced clear operational definitions for each theme and subtheme. (6) Producing the report: a final thematic map and narrative were generated, supported by summary tables that align each component across studies. To illustrate the coding pathway, an example is provided in Table 2.

**TABLE 2:** Sample of coding pathway.

Data extract	Initial code(s)	Subtheme	Theme
Patients in the prehabilitation group received whey protein powder (Inerish; Sino-American Medical Institute Inc, San Diego, CA) daily to achieve adequate protein intake, recommended as 1.5 g/kg/d. The protein supplement was ingested within 1 h after exercise to facilitate muscle synthesis	Whey protein supplement; protein target; protein timing	Protein prescription	Nutritional counselling

## 3 Results

### 3.1 Study characteristics

The 19 articles included in this study were published between 2016 and 2025, with eight conducted in China (Lai et al., 2017a; Lai et al., 2017b; Yang et al., 2018; Liu et al., 2020; Huang et al., 2024; Wang et al., 2020; Chen et al., 2023; Liu et al., 2024), 2 in Canada (Ferreira et al., 2021a; Ferreira et al., 2021b), 1 in Denmark (Sommer et al., 2016), 2 in Spain (Sebio et al., 2017; Fernández-Blanco et al., 2023), 1 in France (Gravier et al., 2022), 1 in Italy (Tenconi et al., 2021), 1 in Portugal (Machado et al., 2024), 1 in Switzerland (Licker et al., 2017), 1 in Turkey (Kökez et al., 2023), and 1 in Czech Republic (Brat et al., 2025). The data extracted from the included articles are organized chronologically in Table 3.

**TABLE 3:** Study characteristics.

Author, year, Place of study	Study design and objectives	Intervention	Results
Sommer et al. (2016) Denmark	Randomized controlled trial To investigate the safety and feasibility of preoperative and early postoperative rehabilitation in a nonhospital setting, with a focus on exercise, in patients undergoing surgery for lung cancer	- Aerobic endurance training	Early postoperative exercise was feasible and safe (no adverse events, most participants completed it). In contrast, the preoperative home-based program was not feasible because diagnostic scheduling and fast-track pathways left only a brief preoperative window
Lai et al. (2017a) China	Randomized controlled trial To investigate short-term preoperative pulmonary rehabilitation combined with inspiratory muscle training and aerobic endurance training in patients scheduled to undergo lung cancer lobectomy	- Aerobic endurance training - Respiratory Muscle training • Abdominal breathing training • Incentive spirometry exercises	A 7-day intensive preoperative pulmonary rehabilitation program yielded greater preoperative improvements in 6-min walk distance and peak expiratory flow, shorter postoperative and total hospital stays, and fewer 30-day postoperative pulmonary complications than no prehabilitation
Lai et al. (2017b) China	Randomized controlled trial To assess the impact of a preoperative 1-week, systematic, high-intensity inpatient exercise regimen on patients with lung cancer who had risk factors for postoperative pulmonary complications	- Respiratory Muscle Training • Abdominal breathing training • Incentive spirometry exercises - Aerobic endurance training	Compared with controls, the intervention group achieved greater gains in 6-min walk distance and peak expiratory flow, shorter total and postoperative length of stay, and fewer postoperative pulmonary complications; short-term rehabilitation was an independent predictor of lower postoperative pulmonary complications risk
Licker et al. (2017) Switzerland	Randomized controlled trial To investigate whether a high-intensity interval training program improves cardiorespiratory fitness before lung cancer surgery and thereby reduces the risk of postoperative complications	- Aerobic endurance training - Resistance training - Preoperative education: healthy nutrition and smoking and alcohol cessation	The intervention group improved peak oxygen consumption and 6-min walk distance, while control group declined in peak oxygen consumption. Overall complication rates did not differ significantly, but pulmonary complications—driven by less atelectasis—were lower in the intervention group, which also had a shorter post-anesthesia care unit stay
Sebio et al. (2017) Spain	Randomized controlled trial To investigate the effects of a preoperative pulmonary rehabilitation program in patients with lung cancer undergoing video-assisted thoracic surgery	- Aerobic endurance training - Resistance training - Respiratory Muscle Training • Incentive spirometry exercises	Prehabilitation led to better preoperative exercise tolerance, higher physical health, and greater muscle strength; no differences were seen immediately after surgery, but by 3 months the prehabilitation group showed greater gains in exercise capacity, physical health, and upper- and lower-limb strength compared with controls
Yang et al. (2018) China	Quasi-experimental trial To evaluate the effect of the self-efficacy-enhancing active cycle of breathing technique on patients with curable lung resection	- Routine perioperative breathing exercise • deep breathing • effective cough exercise - Respiratory Muscle Training • Self-efficacy-enhancing active cycle	Compared with controls, the intervention group had greater sputum in the early postoperative period (days 2–3), lower hospital costs, higher exercise self-efficacy, better 6-min walk performance, and shorter oxygen supplementation duration; no differences were observed in postoperative pulmonary complications or postoperative length of stay
Liu et al. (2020) China	Randomized controlled trial To investigate the impact of a short-term, home-based, multimodal prehabilitation program on perioperative functional capacity in patients undergoing video-assisted thoracoscopic surgery lobectomy	- Aerobic endurance training - Resistance training - Respiratory Muscle training • Incentive spirometry exercise • Cough training • Blow up a small balloon and hold - Nutritional counseling; whey protein supplementation - Psychological adjustment - Preoperative education: perioperative drug recommendations for chronic diseases, smoking cessation, and abstinence - Follow-up weekly	Prehabilitation produced higher perioperative 6-min walk distance and a modest increase in forced vital capacity, with no differences in other lung function measures, disability, psychological outcomes, length of stay, short-term recovery, postoperative complications, or mortality
Huang et al. (2024) China	Randomized controlled trial To investigate the effect of providing breathing exercises to patients with non-small cell lung cancer receiving surgical treatment	- Respiratory Muscle Training • Abdominal breathing training • Pursed-lips breathing • Incentive spirometry exercise • Blow balloon training - Preoperative education: routine pre-and post-surgery care included smoking cessation and abstinence, relevant examinations, and arrangements	Participants in the intervention group showed higher inspiratory capacity and longer 6-min walk distance after preoperative breathing exercises, better inspiratory capacity and less dyspnoea on postoperative day 1, and—at discharge—less dyspnoea, higher inspiratory capacity, and lower anxiety and depression than controls
Ferreira et al. (2021a) Canada	Randomized controlled trial To investigate, in lung cancer patients awaiting elective surgery, the feasibility of delivering a novel 4-week multimodal prehabilitation intervention and its effects on preoperative functional capacity and health-related quality of life, compared to standard hospital care	- Aerobic endurance training - Resistance training - Nutritional counseling: supplementation with whey protein, leucine, vitamin D, EPA and DHA - Psychological adjustment - Preoperative education: healthy diet, physical activity (without specific information), and smoking cessation	The prehabilitation group showed a non-significant trend toward better preoperative 6-min walk test, and no differences between-group were observed in health-related quality of life
Ferreira et al. (2021b) Canada	Randomized controlled trial To determine whether a multimodal prehabilitation program enhances postoperative functional recovery compared with multimodal rehabilitation	- Aerobic endurance training - Resistance training - Nutritional counseling: whey protein supplementation - Relaxation program	Functional capacity was similar between the two multimodal programs at all perioperative time points; by 8 weeks, both groups returned to baseline, with a comparable majority of patients recovered
Tenconi et al. (2021) Italy	Randomized controlled trial To establish whether intensive pulmonary rehabilitation, preoperative and postoperative, improves exercise capacity in patients undergoing lung resection	- Preoperative education: Pain control strategies, self-care and • breathing exercise • sputum clearance technique - Aerobic endurance training - Resistance training - Respiratory Muscle Training • Breathing pattern training • Positive Expiratory Pressure bottle training • Inspiratory Muscle Training	Compared with standard care, pulmonary rehabilitation produced higher exercise tolerance at 6 months and a smaller decline at 1 month postoperatively; no other between-group differences were significant
Fernández-Blanco et al. (2023) Spain	Randomized controlled trial To analyze air leakage and postoperative pain	- Resistance training - Respiratory Muscle Training • Directed breathing • Incentive Spirometry • Expiratory flow increase • Wound protection in coughing - Preoperative education	Compared with the control group, the experimental group had less postoperative air leak during the early postoperative period—while performing physiotherapy techniques, during meals, and during self-directed exercises—with additional reductions during gait later in the early period and a faster decline in the number of patients affected. Pain was lower in the experimental group; the control group reported greater pain across the first four postoperative days and after physiotherapy (except on day 2)
Gravier et al. (2022) France	Randomized controlled trial Identify the effect of condensing 15 prehabilitation sessions into a 3-week regimen compared to a 5-week regime	- Aerobic endurance training - Resistance training - Respiratory muscle training • Positive Expiratory Pressure bottle training - Preoperative education	Compared with the control regimen, the experimental regimen produced similar or modestly greater improvements in V̇O_2_ peak, V̇E/V̇CO_2_ slope, and work rate at the ventilatory threshold; similar effects on peak work rate, V̇O_2_ at the ventilatory threshold, body mass index, and maximal inspiratory pressure; and uncertain effects on quadriceps strength, quality of life, and postoperative complications
Wang et al. (2020) China	Randomized controlled trial To assess the effectiveness of a rapid and precise pulmonary rehabilitation nursing program during the perioperative period	- Personalized breathing training (activities are not described, but were provided with instructions about the exercises)	The experimental group showed better pulmonary function, a shorter hospital stay, and higher quality of life than controls (differences not statistically significant), while the postoperative complication rate was significantly higher in the control group
Kökez et al. (2023) Turkey	Randomized controlled trial To investigate the effects of the preoperative short term intensive pulmonary rehabilitation program applied for patients who have undergone lung resection by thoracotomy, on lung functions, complication rates and length of hospital stay during the postoperative period	- Respiratory Muscle Training • Abdominal breathing exercises • Segmental breathing exercises (unilateral, posterior, bilateral basal, and apical) • Puckered lip breathing • Incentive Spirometer • Coughing technique - BIPAP application - Preoperative education	Compared with the study group, the control group had a higher overall complication rate and, among patients undergoing lobectomy or wedge resection, a longer hospital stay (both statistically significant)
Chen et al. (2023) China	Randomized controlled trial To evaluate the effects of mindful breathing training combined with diary-based rehabilitation guidance on improving perioperative outcomes in lung cancer surgery patients	- Mindful Breathing - Preoperative education	Participants assigned to mindful breathing showed statistically significant improvements in dyspnea, fatigue and anxiety
Machado et al. (2024) Portugal	Randomized controlled trial To investigate the effect of preoperative home-based exercise training on quality of life after lung cancer surgery	- Aerobic endurance training - Resistance training - Preoperative education - Telephone-based supervision	Participants in the intervention group showed higher global quality of life than controls before and after surgery (statistically significant), less pain and appetite loss, better postoperative physical, emotional, and role functioning, and superior performance on preoperative five-times sit-to-stand and postoperative exercise capacity compared with the control group
Chen et al. (2023) China	Randomized controlled trial To analyze the effect of an exercise-nutrition management model based on the ERAS concept on patients undergoing thoracoscopic radical surgery for lung cancer	- Respiratory Muscle Training • Abdominal breathing exercises • Puckered lip breathing • Coughing technique • Blow balloon training - Preoperative education - Psychological adjustment - Nutritional counseling	Participants in the intervention group had lower postoperative pain (days 2–3), higher medication adherence, better nutritional status, better pulmonary function, less fatigue and dyspnoea, and higher health-related quality of life than controls; complication rates did not differ significantly
Brat et al. (2025) Czech Republic	Randomized controlled trial To evaluate whether a 14-day multimodal prehabilitation program reduces postoperative pulmonary and cardiovascular complications and hospital length of stay in high-risk patients undergoing elective lung resection	- Respiratory Muscle Training • Incentive spirometer - Psychological adjustment - Preoperative education: smoking cessation - Nutritional counseling	Participants allocated to multimodal prehabilitation had fewer postoperative pulmonary complications, a shorter hospital stay, a reduction in VE/VCO_2_ slope after the program, and better patient-reported quality of life compared with usual care

**FIGURE 2:**
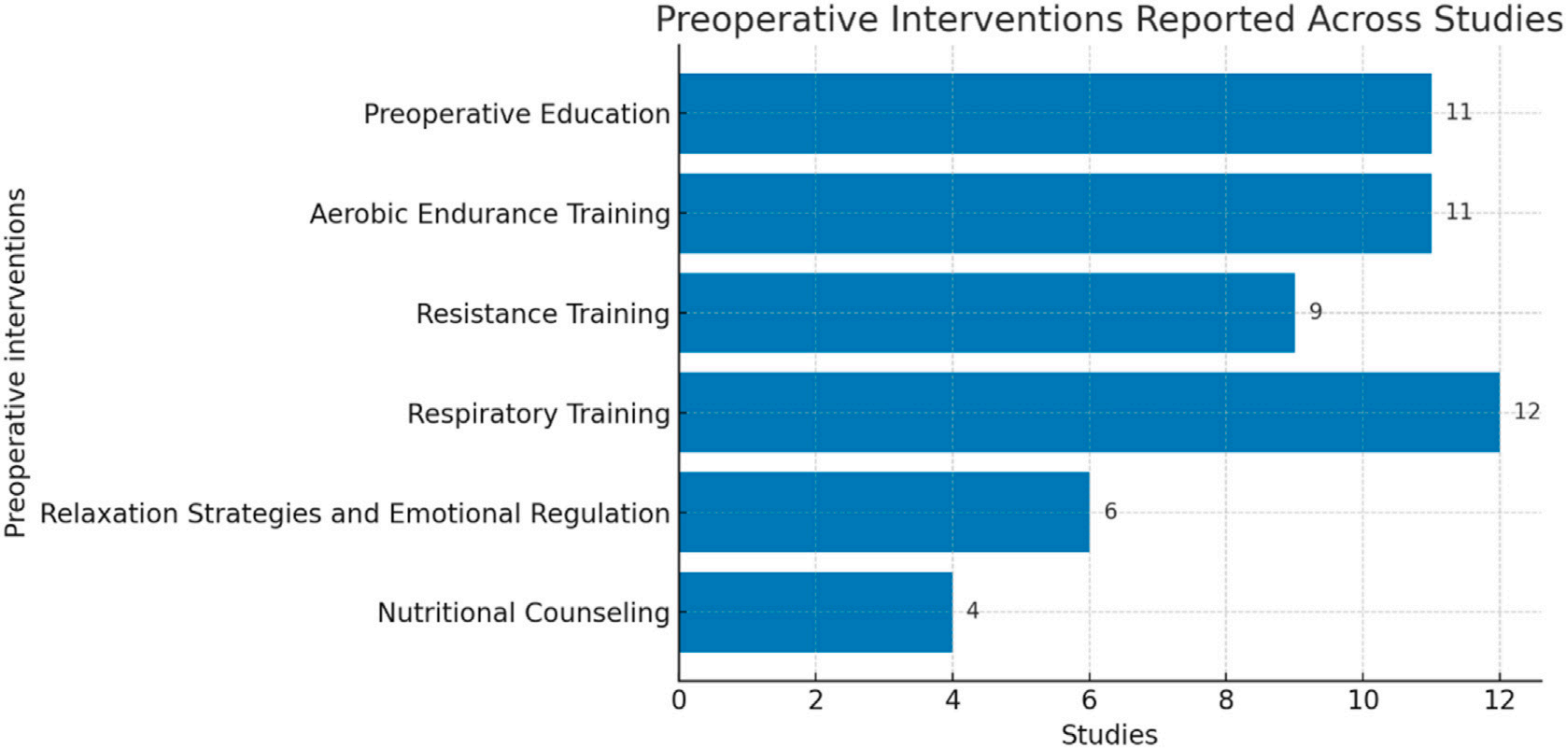
Preoperative interventions.

Only the interventions performed in the preoperative period were included in this review. The interventions were grouped into categories as shown in Figure 2, which identifies the number of articles that mention each intervention.

### 3.2 Preoperative education

Regarding preoperative education, several studies (Liu et al., 2020; Huang et al., 2024; Chen et al., 2023; Liu et al., 2024; Ferreira et al., 2021a; Fernández-Blanco et al., 2023; Kökez et al., 2023; Brat et al., 2025) incorporated training and respiratory exercises. During this contact, pre-and postoperative routines, such as scheduling and complementary diagnostic exams, were discussed (Huang et al., 2024; Chen et al., 2023) and the stages of the surgical process (Fernández-Blanco et al., 2023; Kökez et al., 2023).

In several studies, teaching was also provided regarding nutritional adjustments, smoking cessation, and alcohol abstinence (Liu et al., 2020; Huang et al., 2024; Chen et al., 2023; Ferreira et al., 2021a; Gravier et al., 2022; Licker et al., 2017; Brat et al., 2025). Furthermore, instructions on the therapeutic management of chronic diseases were provided (Liu et al., 2020; Huang et al., 2024). Through this intervention, participants were encouraged to engage in physical activity, although without specific or directive instructions (Ferreira et al., 2021a).

At this stage, the effectiveness of respiratory exercises is reinforced, with encouragement to perform them. Education was combined with exercise practice, consolidating teachings on deep breathing, airway clearance, cough with wound containment, and postoperative mobilization (Chen et al., 2023; Gravier et al., 2022; Machado et al., 2024; Kökez et al., 2023). Additionally, brochures with descriptions of exercises and illustrative images were provided, along with activity logbooks for use at home.

It is worth noting that while there was no formal educational component in the study by Yang et al. (2018), a leaflet was distributed (Yang et al., 2018; Ferreira et al., 2021a; Ferreira et al., 2021b; Fernández-Blanco et al., 2023).

Although (Liu et al., 2024) did not provide a detailed syllabus of the educational content, the intervention used literacy-sensitive, face-to-face communication and multimodal delivery (technique demonstrations, video playback, and a WeChat mini-program) to educate patients and family members. Teaching was organized in stages and scheduled over the perioperative timeline.

### 3.3 Aerobic endurance training

Eleven of the sixteen studies identified aerobic endurance training as an integral part of the preoperative program (Lai et al., 2017a; Lai et al., 2017b; Liu et al., 2020; Ferreira et al., 2021a; Ferreira et al., 2021b; Sommer et al., 2016; Sebio et al., 2017; Gravier et al., 2022; Tenconi et al., 2021; Machado et al., 2024; Licker et al., 2017). There were a variety of devices used in this type of intervention. Some studies (Lai et al., 2017a; Lai et al., 2017b; Liu et al., 2020; Sebio et al., 2017; Gravier et al., 2022; Licker et al., 2017) incorporated devices such as the elliptical bike, stationary bike, ergonomic bike, or cycle ergometer, while others used walking, jogging, or cycling (Liu et al., 2020; Ferreira et al., 2021a; Ferreira et al., 2021b; Tenconi et al., 2021; Licker et al., 2017). The exercise choice varied according to the patient’s individual preference, particularly when not supervised. One study (Sommer et al., 2016) did not report the type of aerobic exercise performed.

Regarding training duration, the most common duration was 30-min training sessions (Lai et al., 2017a; Lai et al., 2017b; Liu et al., 2020; Ferreira et al., 2021a; Sebio et al., 2017; Tenconi et al., 2021). However, across other studies, training durations ranged from 15 to 45 min, with some protocols starting shorter and gradually increasing over time (Gravier et al., 2022; Machado et al., 2024; Licker et al., 2017).

Training adjustments were made according to the individual’s capabilities (Lai et al., 2017a; Lai et al., 2017b; Liu et al., 2020; Ferreira et al., 2021a; Ferreira et al., 2021b; Sommer et al., 2016; Sebio et al., 2017; Gravier et al., 2022; Tenconi et al., 2021; Machado et al., 2024; Licker et al., 2017). Intensity was adjusted according to the individual’s perceived effort, assessed using the Borg or modified Borg scale (Lai et al., 2017a; Lai et al., 2017b; Liu et al., 2020; Ferreira et al., 2021a; Sebio et al., 2017; Machado et al., 2024). However, in the studies by Licker et al. (2017) and Liu et al. (2020), intensity was also determined by using target heart rate. Additionally, Ferreira et al. (2021a), established a training load in watts based on an incremental test limited by symptoms. The authors' exercise intensity prescriptions are organized in Table 4.

**TABLE 4:** – Exercise intensity prescriptions.

Author	Intensity
Sommer et al. (2016)	Warm-up 5 min to ∼85% HRmax; then 25 min of 1–2 min intervals at 85%–100% HRmax with 1-min rests; cool-down 2 min
Lai et al. (2017a)	Not reported
Lai et al. (2017b)	Not reported
Licker et al. (2017)	Warm-up 5 min at 50% peakWR; 2 × 10 min of 15-s sprints at 80%–100% peakWR with 15-s pauses and 4-min inter-set rest; cool-down 5 min at 30% peakWR.
Sebio et al. (2017)	Interval: 30 min total — cycles of 1 min at 80% Wpeak +4 min at 50% Wpeak; includes warm-up 5 min and cool-down 4 min at 30% Wpeak
Liu et al. (2020)	Moderate–high intensity by RPE 13–16 (Borg 6–20) and target HR ≈ 70% HRR: (220−age−resting HR)×0.70 + resting HR.
Ferreira et al. (2021a)	90% of workload at anaerobic threshold (cardiopulmonary exercise test baseline) on cycle ergometer, 30 min
Ferreira et al. (2021b)	Not reported
Tenconi et al. (2021)	60%–80% HRmax
Gravier et al. (2022)	Cycling workload increased by 5–10 W as tolerated (target intensity not specified)
Machado et al. (2024)	Duration progression from 30 min (week 1) to 40 min (week 2+); intensity not reported

Abbreviations: HRmax, maximal heart rate; peakWR/Wpeak = peak work rate; HRR, heart-rate reserve; RPE, rating of perceived exertion.

It is also important to note that Licker et al. (2017), Sebio et al. (2017), Liu et al. (2020), Ferreira et al. (2021a), and Gravier et al. (2022) described this type of training as cyclical, including warm-up, exercise, and cool-down, with intensity adjustments at each stage of the intervention. The warm-up and cool-down stages each lasted for 5 min across studies.

### 3.4 Resistance training

Of the sixteen studies included in the investigation, nine (Liu et al., 2020; Ferreira et al., 2021a; Ferreira et al., 2021b; Sebio et al., 2017; Fernández-Blanco et al., 2023; Gravier et al., 2022; Tenconi et al., 2021; Machado et al., 2024; Licker et al., 2017) identified strength training as a potentiator in the preoperative phase. Across studies, this type of training involved different exercises; however, authors were not specific about the preferred exercises. The exception is (Licker et al., 2017), who describe exercises such as “leg press, leg extension, back extension, seated row, biceps curls,” or “chest and shoulder press,” and (Gravier et al., 2022), who describe “leg extension, arm pull-down,” and “arm extension.”

Execution of resistance training involved the use of various materials, such as machines and elastic bands (Liu et al., 2020; Ferreira et al., 2021a; Ferreira et al., 2021b; Sebio et al., 2017; Gravier et al., 2022), body weight (Ferreira et al., 2021a; Sebio et al., 2017), or free weights, including weights or dumbbells (Ferreira et al., 2021a). However, the most used material among studies was elastic bands, with varying resistance levels adjusted to everyone’s capabilities.

In most studies (Liu et al., 2020; Ferreira et al., 2021a; Sebio et al., 2017; Tenconi et al., 2021; Licker et al., 2017), training sessions focused on the main muscle groups (back, chest, upper and lower limbs). In contrast, Fernández-Blanco et al. (2023), Gravier et al. (2022), and Machado et al. (2024) identified only certain muscle groups, primarily focusing on the peripheral muscles of the upper and lower limbs.

The authors' recommendations for resistance training—specifically frequency, intensity, and volume—are organized in Table 5. Licker et al. (2017) and Fernández-Blanco et al. (2023) did not describe the exercise plan implemented in their studies.

**TABLE 5:** Resistance training recommendation.

Author	Frequency of resistance training	Volume	Intensity
Sebio et al. (2017)	3 to 5 times per week	3 sets 15 repetitions	Increase to 4 sets after 10 weeks. Recommended intensity, score 4–7, moderate (OMNI scale)
Liu et al. (2020)	2 times per week	3 sets 10–12 repetitions	Recommended intensity, score 13–16, moderate (Borg scale)
Ferreira et al. (2021a)	3 times per week	1–2 sets 8–15 repetitions	Recommended to increase intensity, score <10, light (Borg scale)
Ferreira et al. (2021b)	3 times per week	2 sets 8–12 repetitions	Not described
Tenconi et al. (2021)	Supervised 2 to 3 times a week Unsupervised 3 to 4 times a week	Not described	Not described
Gravier et al. (2022)	5 times per week OR 3 times per week	3 sets 12 repetitions	60%–70% of 1 RM. Increase according to tolerance
Machado et al. (2024)	2 times per week	2 sets 15 repetitions	Increase to 3 sets after 2 weeks. Recommended intensity, score 3–5, moderate to intense (modified Borg scale)

Studies have also reported that stretching exercises were performed (Liu et al., 2020; Ferreira et al., 2021a; Ferreira et al., 2021b).

### 3.5 Respiratory training

The study by Wang et al. (2020) reported the implementation of personalized respiratory training but did not specify the exercises performed. Respiratory training was provided in person, with sessions lasting 30 min over 3 weeks, although the frequency of daily sessions was not mentioned in the study.

However, other studies subdivided respiratory training interventions into two categories based on their descriptions, which were complemented using BiPAP, Functional Respiratory Reeducation and Inspiratory Muscle Training.

#### 3.5.1 Functional Respiratory Reeducation

Concerning the interventions included in the Functional Respiratory Reeducation category, nine studies (Lai et al., 2017a; Lai et al., 2017b; Yang et al., 2018; Liu et al., 2020; Huang et al., 2024; Sebio et al., 2017; Fernández-Blanco et al., 2023; Tenconi et al., 2021; Kökez et al., 2023) referenced them in the preoperative period. The interventions mentioned in this category include awareness and respiratory control (Yang et al., 2018; Tenconi et al., 2021), diaphragmatic breathing (Lai et al., 2017b; Yang et al., 2018; Huang et al., 2024; Fernández-Blanco et al., 2023; Kökez et al., 2023), coastal reeducation (Kökez et al., 2023), incentive spirometry (Lai et al., 2017a; Lai et al., 2017b; Liu et al., 2020; Sebio et al., 2017; Fernández-Blanco et al., 2023; Kökez et al., 2023), training with balloon blowing (Liu et al., 2020; Huang et al., 2024; Liu et al., 2024), and expiration with pursed lips (Huang et al., 2024; Liu et al., 2024; Kökez et al., 2023). Additionally, mechanisms for clearing the airways, such as specifically directed cough (Yang et al., 2018; Liu et al., 2020; Kökez et al., 2023), active cycle of respiratory techniques (Yang et al., 2018), and cough with wound containment (Liu et al., 2024; Fernández-Blanco et al., 2023), were included.

The awareness and respiratory control interventions did not specify the frequency of performance. The training included these interventions, which were reported to be carried out both in the rehabilitation center and at home (Yang et al., 2018; Fernández-Blanco et al., 2023; Tenconi et al., 2021). In the studies by Lai et al. (2017a) and Lai et al. (2017b), diaphragmatic breathing was performed twice a day, with a duration between 15 and 30 min, sitting or in dorsal decubitus, with the knees bent and the shoulders relaxed, with the guidance and supervision of a trained professional. Kökez et al. (2023) reported performing 10 repetitions per day for 7 days at home, as well as conducting the exercises at the rehabilitation center. Costal reeducation was only performed in the study by (Kökez et al., 2023), and it was conducted laterally, posteriorly, basally, apically, and globally, with 10 repetitions per day at the rehabilitation center.

An incentive spirometer was performed at the rehabilitation unit, including deep breathing exercises with active inspiration, breath retention, and passive expiration. This was done thrice daily for 20 repetitions (Lai et al., 2017a; Lai et al., 2017b). However, (Sebio et al., 2017), performed it twice daily, at 80% of the maximum vital capacity (measured previously), with an inspiratory pause, completing six full and five repetitions, with 1 minute of rest between cycles. (Kökez et al., 2023). performed 15 repetitions per day. In the balloon-blowing training, (Liu et al., 2020), and (Huang et al., 2024) refer to it, with Liu et al. stating that the balloon is inflated in one breath and held for more than 5 seconds. Huang et al. (2024) and Kökez et al. (2023) mentioned expiration with pursed lips, performed for 10 repetitions per day.

The airway clearance mechanism encompassed different strategies, which were mentioned by Yang et al. Yang et al. (2018), Liu et al. (2020), and Kökez et al. (2023) as directed cough, performed both at home and at the rehabilitation center. The Active Cycle of Breathing Techniques (ACBT) in the study by Yang et al. (2018), consisted of the forced expiration technique (huffing) with breath control. They reported that it should be performed comfortably, either sitting or reclining, with three to five repetitions as tolerated, lasting 15–20 min. It was also mentioned that additional cycles should be performed if the person feels secretions in the upper airways. Although this was done at home in the study, it was initially performed in person with groups of three to five people (Yang et al., 2018).

Finally, cough with wound containment was mentioned exclusively by Fernández-Blanco et al. (2023) as a training component. However, other studies presented it as a preoperative education strategy (Ferreira et al., 2021b; Gravier et al., 2022).

Not all authors were descriptive regarding the time and repetitions; instead, as they only referred to the total time or repetitions encompassing the set of interventions they introduced.

#### 3.5.2 Inspiratory muscle training

Respiratory Muscle Training was referenced in two studies (Gravier et al., 2022; Tenconi et al., 2021; Brat et al., 2025). Pressure-type training was performed using the Threshold IMT–Breathing Trainer (Phillips^®^) device, with at least 30% of the maximum inspiratory pressure. Participants were encouraged to perform 15 min of training, with the recommendation to increase the resistance (Gravier et al., 2022) regularly (Brat et al., 2025) employed the same device twice daily (20 min morning and 20 min afternoon) over 2 weeks. Phase 1 (week 1) prescribed a constant load at 50% of baseline maximal inspiratory pressure/maximal expiratory pressure, when expiratory training was undertaken). In Phase 2 (week 2), maximal inspiratory pressure/maximal expiratory pressure was reassessed before supervised sessions, and the load was increased to 60%. Tenconi et al. (2021) also reported inspiratory training but did not provide exercise parameters or session duration.

#### 3.5.3 BiPAP

In the study by Kökez et al. (2023), in addition to the previous interventions, the application of BiPAP (Bi-level Positive Airway Pressure) for 20 min per day was also part of their intervention, aimed at improving ventilation. However, the specific ventilation parameters applied were not specified.

### 3.6 Relaxation strategies and emotional regulation

Interventions in this category, reported across several studies (Liu et al., 2020; Chen et al., 2023; Liu et al., 2024; Ferreira et al., 2021a; Ferreira et al., 2021b; Brat et al., 2025), aimed to optimize psychological wellbeing. Three studies (Liu et al., 2020; Ferreira et al., 2021a; Ferreira et al., 2021b) taught mental relaxation techniques (visualization, guided imagery, and deep/diaphragmatic breathing), often accompanied by relaxing music. The frequency of practice varied: Chen et al. (2023) prescribed daily sessions before bedtime, whereas (Ferreira et al., 2021a; Ferreira et al., 2021b) delivered these techniques in person two to three times per week during clinic visits. Brat et al. (2025) reported breathing relaxation techniques but did not provide parameters. Liu et al. (2024) stated that participants exhibiting negative emotions (e.g., anxiety or concerns) received psychological counselling, without detailing the content of that intervention.

Chen et al. (2023) presented mindfulness as an additional strategy. Participants received mindfulness training from the first day of participation until the day before surgery. Each session, planned for 15 min, was conducted twice a day according to audio instructions provided. The technique involved choosing a quiet environment, with the person in a comfortable position, either lying down or sitting. The person was instructed to take two slow, deep breaths, concentrating on the sensation of the abdomen expanding with each inhalation and contracting with each exhalation.

### 3.7 Nutritional counseling

Nutritional counseling was mentioned in four studies (Liu et al., 2020; Liu et al., 2024; Ferreira et al., 2021a; Ferreira et al., 2021b). According to Liu et al. (2020), dietary adjustments were made following a nutritional assessment using a 3-day food diary. These adjustments aimed to improve eating habits by reducing excess calories, increasing the intake of vegetables and fruits, and consuming high-quality protein. Whey protein (1.5 g/kg/day) was also introduced 1 hour after exercise to enhance muscle synthesis.

Ferreira et al. (2021a) also emphasized the importance of nutritional assessment and introduced whey protein in pre-prepared doses of 10 or 20 g, taken twice daily. Additionally, 3 g of leucine were added to each protein dose, mixed in 125 mL of water. Participants were also instructed to take a 10 mL dose of fish oil, which contained vitamin D3 (2000 IU), DHA (1000 mg), and EPA (1500 mg). Ferreira et al. (2021b) reported that nutritional assessment was carried out using the Patient-Generated Subjective Global Assessment (PG-SGA) and Nutritional Risk Screening (NRS 2002) scales, along with a 3-day food diary. Liu et al. (2024) likewise used the PG-SGA and had a dietitian develop an individualized nutrition plan based on the assessment results, patient preferences, and clinical status. Brat et al. (2025) screened all prehabilitation participants with the Malnutrition Universal Screening Tool (MUST); those with a MUST score ≥2 were referred to the nutrition support team. Another study (Ferreira et al., 2021a) suggested ingesting whey protein 1 hour after exercise.

## 4 Discussion

This scoping review systematically mapped preoperative prehabilitation interventions in adults with lung cancer undergoing thoracic surgery, detailing what is delivered and how it is delivered. The included studies most often described multimodal programs combining aerobic endurance training and resistance training with respiratory training (Lai et al., 2017a; Lai et al., 2017b; Liu et al., 2020; Ferreira et al., 2021a; Ferreira et al., 2021b; Sebio et al., 2017; Licker et al., 2017). In contrast, Yang et al. (2018), Huang et al. (2024), Wang et al. (2020), and Kökez et al. (2023) referred only to respiratory exercise as a training component.

Within these programs, aerobic endurance training aims to increase aerobic capacity by improving the cardiovascular, respiratory, and musculoskeletal systems (Machado et al., 2021). On the other hand, resistance training focuses on improving muscle contraction against external resistance, enhancing muscular endurance (Machado et al., 2021). Respiratory training aims to increase maximum inspiratory pressure, helping to control dyspnea and improve alveolar ventilation (Ordem dos Enfermeiros, 2018).

Regarding intensity prescription, most authors (Lai et al., 2017a; Lai et al., 2017b; Wang et al., 2020; Chen et al., 2023; Ferreira et al., 2021b; Sebio et al., 2017; Gravier et al., 2022; Machado et al., 2024) used the relationship between perceived effort and the load applied, employing the Borg scale, which is a valid and reliable indicator for monitoring exercise tolerance (Machado et al., 2021). However, in the study by Licker et al. (2017), it was determined that exercise power using power meters in watts allowed for measuring instantaneous changes and controlling effort more specifically (Robinson et al., 2011). This type of device is costly, which was reported as a limitation. When compared to measuring intensity by heart rate, Robinson et al. (2011) argued that there were no significant advantages in using power meters for average recreational performance, suggesting that low-cost heart rate monitors are equally capable of functioning as training monitoring devices.

The respiratory training interventions (Lai et al., 2017a; Huang et al., 2024; Sebio et al., 2017; Fernández-Blanco et al., 2023; Tenconi et al., 2021; Kökez et al., 2023) aligned with the goals of respiratory functional reeducation (Dias et al., 2022). These goals include improving ventilation and lung re-expansion, clearing the airways through the mobilization and expulsion of secretions, enhancing oxygenation and gas exchange, increasing thoracic mobility, re-educating respiratory muscles, and boosting muscular strength and endurance. These effects contribute to the prevention of complications and promote pulmonary recovery. Notably, Fernández-Blanco et al. (2023), mentioned aerosol sessions, although they did not specify the rationale or outcomes. Additionally, Kökez et al. (2023) noted the application of BiPAP within the scope of the preoperative rehabilitation program, which was aimed at improving pulmonary mechanics.

Consistent with prior reviews (Granger and Cavalheri, 2022; Edbrooke et al., 2023; Voorn et al., 2023), the evidence supports prehabilitation as beneficial, yet substantial heterogeneity limits firm guidance on the optimal duration, intensity, structure, and patient selection. This heterogeneity spans procedure, program design, exercise prescription and outcome definitions. Together, these differences likely dilute pooled effects and make cross-study comparisons difficult.

Relaxation strategies and emotion-regulation interventions were identified across several studies (Liu et al., 2020; Chen et al., 2023; Liu et al., 2024; Ferreira et al., 2021a; Ferreira et al., 2021b; Brat et al., 2025), Mindfulness was examined by Liu et al. (Chen et al., 2023), who compared a stand-alone conscious breathing protocol with a combined approach that also included rehabilitation guidelines. Although the rehabilitation guidelines were not specified, both approaches demonstrated a reduction in anxiety, improvement in emotional resilience, and optimization of postoperative recovery. However, the combined group was not more effective than the isolated group. This type of training allowed for establishing respiratory rhythms, regulating respiratory disturbances, and improving gas exchange.

These observations are consistent with previous syntheses, which note that many lung-cancer prehabilitation packages include psychoeducational and anxiety-reduction components—for example, guided breathing/relaxation, mindfulness, or brief coping skills—although reporting of dose and delivery is often limited (Edbrooke et al., 2023). This is likely important because preoperative anxiety is common and prognostically relevant, being associated with higher postoperative pain, poorer quality of life, and longer recovery; brief psychological modules are low-cost, feasible in short preoperative windows, and may enhance adherence to exercise and nutrition. Consequently, embedding a minimum psychological bundle (clear education plus a simple relaxation/breathing routine and basic coping guidance) within multimodal prehabilitation is justified, while future trials should specify content and dose, monitor fidelity, and test mediators such as anxiety or self-efficacy to clarify mechanisms of benefit.

Nutrition remains under-reported in operational terms despite its prominence in ERAS (Batchelor et al., 2019). Malnutrition and/or preoperative weight loss are important predictors of postoperative complications (Batchelor et al., 2019; Asakawa et al., 2024; Campbell et al., 2023). Accordingly, nutritional and rehabilitation interventions, the goal is to reduce the incidence of postoperative complications and improve prognosis (Asakawa et al., 2024; Campbell et al., 2023). Where specified, teams used screen-and-treat pathways that triggered targeted supplementation and individualized dietetic plans. Liu et al. (2020), Ferreira et al. (2021a), and Gravier et al. (2022) incorporated formal nutritional assessment with supplement adjustments as indicated. Liu et al. (2020) recommended the intake of whey protein at 1.5 g/kg/day, which aligns with recommendations from other international studies; however, the dosage should be tailored to the individual’s needs, based on prior assessment, with a range of 1.0–1.6 g/kg/day (Asakawa et al., 2024; Campbell et al., 2023; Deutz et al., 2014).

The importance of preoperative education was also emphasized in previous studies (Mendes et al., 2021) stated that through preoperative nursing consultations, change is promoted by improving processes and outcomes. This leads to better preparation, more information, and greater collaboration. Several studies also mentioned this practice as part of the rehabilitation process (Liu et al., 2020; Huang et al., 2024; Chen et al., 2023; Ferreira et al., 2021a; Fernández-Blanco et al., 2023; Kökez et al., 2023), an integral component of the preoperative plan.

It is also noteworthy that several studies (Liu et al., 2020; Huang et al., 2024; Chen et al., 2023; Ferreira et al., 2021a; Gravier et al., 2022; Licker et al., 2017) advocated for smoking cessation and alcohol abstinence, which aligns with international recommendations (Mendes et al., 2018). These guidelines emphasize that both alcohol consumption and smoking are associated with increased morbidity and mortality risk and should be discontinued, ideally 4 weeks before surgery (Batchelor et al., 2019).

The analysis of the selected articles also demonstrates that prehabilitation plans can be implemented at the rehabilitation center and home or even simultaneously. Considering the assumptions of the training, it is understood that the included studies used their interventions to improve functional capacity and aerobic function, as well as to reduce fatigue (Lai et al., 2017a; Liu et al., 2020; Chen et al., 2023; Sebio et al., 2017; Fernández-Blanco et al., 2023; Tenconi et al., 2021; Licker et al., 2017). Furthermore, studies by Lai et al. (2017b), Tenconi et al. (2021), Sommer et al. (2016), and Kökez et al. (2023) demonstrated a reduction in postoperative complications. In the interventions established by Lai et al. (2017a), Lai et al. (2017b), Fernández-Blanco et al. (2023), and Kökez et al. (2023), a reduction in hospitalization time was demonstrated, as well as a decrease in hospital costs (Lai et al., 2017b; Yang et al., 2018). In addition to physical benefits, studies identified improvements in both quality of life (Lai et al., 2017a; Wang et al., 2020; Gravier et al., 2022; Machado et al., 2024) and a reduction in anxiety (Liu et al., 2020; Huang et al., 2024; Chen et al., 2023; Machado et al., 2024). However, Sommer et al. (2016) reported no benefits from their preoperative intervention. This was due to the reduced sample size and the fact that the program lasted 4 weeks, while the country’s guidelines required people to be operated on within 2 weeks, making it unfeasible to meet the proposed timeframe.

Overall, our map of preoperative prehabilitation components accords with prior syntheses showing benefit signals for prehabilitation in lung cancer while highlighting operational details that earlier reviews did not emphasize. Previous reviews also concluded that prehabilitation is promising yet heterogeneous, making optimal duration, intensity, structure, and patient selection uncertain. Our findings complement these conclusions by describing how interventions have been delivered in recent trials, thereby addressing a recognized gap in implementation-oriented reporting.

This review has clear implications for clinical practice. It shows that prehabilitation should be included as a routine part of care for individuals with lung cancer undergoing thoracic surgery. The evidence suggests combining physical training (aerobic endurance, resistance, and respiratory training), nutritional support, preoperative education, and relaxation/emotion-regulation can improve outcomes. This means developing personalized exercise programs to increase physical capacity, offering preoperative consultations to reduce anxiety and improve patient engagement, and assessing nutritional needs with appropriate supplementation. It is also recommended to support smoking cessation and alcohol abstinence ideally 4 weeks before surgery. Psychological support, including breathing training and mindfulness, should be considered to reduce anxiety and support emotional resilience. Depending on patients’ needs and available time, these interventions can be implemented at hospital, at home, or both. Applying these measures in a structured and interdisciplinary way can help reduce postoperative complications, shorten hospital stays, and improve recovery and quality of life.

### 4.1 Strengths and limitations

The strengths of this review are that it primarily focuses on randomized studies, which allows for a transparent and targeted investigation of the interventions carried out during the preoperative period in thoracic surgery. This investigation also identified interventions previously studied in practical and experimental contexts and their techniques and methods. Additionally, it contributes to strengthening evidence-based practices, ultimately improving the performance of the rehabilitation nurse specialist. Limitations included studies exclusively published only in English and Portuguese were included, which may have overlooked valuable information in other languages. As well, the fact that not all studies provide detailed descriptions of the interventions limited the ability to understand some of them entirely.

## 5 Conclusion

Practical and tailored prehabilitation protocols can reduce postoperative complications, shorten the length of stay, and consequently lower associated costs, ultimately improving survival outcomes in treating the disease. This is an important area for future research, aiming at developing and modifying programs and protocols. This review sought to identify which prehabilitation interventions applicable in the preoperative period of thoracic surgery could provide more significant benefits to the therapeutic process and enhance long-term quality of life after the completion of this treatment modality.

The recommendations from this review are that rehabilitation programs should encompass both aerobic endurance training and resistance training, as well as respiratory training, including functional respiratory rehabilitation and inspiratory muscle training. As well, preoperative education is a key component, with the encouragement of alcohol abstinence and smoking cessation serving as a cornerstone. Nutritional counseling and relaxation/emotion-regulation strategies should also be considered, aligning with international guidelines, where personalization is essential to make the intervention individualized. With the knowledge synthesis in this review, rehabilitation nurse specialists can establish prehabilitation intervention plans aimed at caring for, empowering, and maximizing patients' potential. However, further research is needed to demonstrate the potential of prehabilitation in postoperative recovery and the prevention and/or reduction of postoperative complications.

## Data Availability

The original contributions presented in the study are included in the article/supplementary material, further inquiries can be directed to the corresponding author.
